# CT Cadaveric dataset for Radiomics features stability assessment in lumbar vertebrae

**DOI:** 10.1038/s41597-024-03191-6

**Published:** 2024-04-11

**Authors:** Riccardo Levi, Maximiliano Mollura, Giovanni Savini, Federico Garoli, Massimiliano Battaglia, Angela Ammirabile, Luca A. Cappellini, Simona Superbi, Marco Grimaldi, Riccardo Barbieri, Letterio S. Politi

**Affiliations:** 1 Neuroradiology Department, IRCCS Humanitas Research Hospital, Via Manzoni 56, Rozzano, 20089 Milan, Italy; 2 Department of Electronic, Information and Bioengineering, Politecnico di Milano, Piazza Leonardo da Vinci 32, 20133 Milan, Italy; 3 Department of Biomedical Sciences, Humanitas University, Via Rita Levi Montalcini 4, Pieve Emanuele, 20072 Milan, Italy

**Keywords:** Bone, Preclinical research

## Abstract

Radiomics features (RFs) studies have showed limitations in the reproducibility of RFs in different acquisition settings. To date, reproducibility studies using CT images mainly rely on phantoms, due to the harness of patient exposure to X-rays. The provided CadAIver dataset has the aims of evaluating how CT scanner parameters effect radiomics features on cadaveric donor. The dataset comprises 112 unique CT acquisitions of a cadaveric truck acquired on 3 different CT scanners varying KV, mA, field-of-view, and reconstruction kernel settings. Technical validation of the CadAIver dataset comprises a comprehensive univariate and multivariate GLM approach to assess stability of each RFs extracted from lumbar vertebrae. The complete dataset is publicly available to be applied for future research in the RFs field, and could foster the creation of a collaborative open CT image database to increase the sample size, the range of available scanners, and the available body districts.

## Background & Summary

The application of artificial intelligence (AI) to image processing techniques in the field of radiology has already demonstrated a plethora of efficacious clinical implementations, resulting in notable advancements in medically cleared, AI-driven devices that have significant implications in clinical practice. AI medical devices mainly rely on automatically extracted features (Deep Learning) to assist radiological diagnosis. Radiomic Features (RFs) were developed to provide quantitative and standardized information about shape, density/intensity, and texture patterns of anatomical or pathological structures from radiological images, but currently very few RF-based software were approved for the use in clinical practice. As a matter of facts, several studies performed on both patients^1^ and phantoms^2^ showed limitations in the standardization of RFs with respect to different acquisition parameters and CT scanners, and appropriate validation on external, freely available research database^3^.

The Image Biomarker Standardization Initiative (IBSI)^4^ addressed these challenges of non-reproducibility by proposing a structured pipeline to strengthen the validity of RFs-based studies. Several algorithms were therefore proposed to improve standardization of RFs across different CT acquisitions and/or CT scanners, (e.g., z-score or ComBat^5^).

To date, reproducibility studies using CT images mainly rely on phantoms^6,7^, to avoid subjecting patients to X-ray exposure. Even though materials are designed to simulate human structure, some works proved that not all materials are stable when CT acquisition protocol changes^8^, and might not represent the complexity of human body structures.

Cadaveric studies could provide a more accurate description of organs’ textural structure. Only a limited number of studies investigated the effects of CT protocols on cadaveric parts of the body^9^, which were acquired outside the donor’s body.

Here we present a thoughtful dataset comprising CT images obtained on a cadaveric thoraco-abdominal trunk. CT images from the same cadaver were acquired with varying CT acquisition parameters (mA, kV, field-of-view, Reconstruction Kernel) on 3 CT scanners from different vendors and with different number of detectors; a proper test-retest procedure was also performed on a single CT scanner, with a total of 112 unique CT acquisitions. Given this extensive dataset, we propose a thorough pipeline to evaluate the effect of each CT acquisition parameter on RF extracted from lumbar vertebrae, together with a quantitative comparison in accuracy of RFs harmonization between Generalized Linear Model (GLM) and the ComBat algorithm. We focused the analysis on vertebrae since there is evidence of maintaining the biomechanical properties also on cadaveric subjects^10,11^, as well as exhibit a trabecular pattern that is highly difficult to be replicable in manufactured phantoms^12^.

The whole image dataset and the GLM model are made available for further analyses, either tailored to other body organs, or to different Radiomics libraries, or to the development of further optimized standardization algorithms. We hope that the presented dataset could integrate the international efforts in standardization of Radiomics features and their translation into clinical practice, as well as paving the way for the development of advance AI-based algorithms for image harmonization on medical images.

## Methods

### Cadaver donor

The human cadaver used in this study belonged to an 80-year-old Caucasian man, and was provided by MedCure Inc. The cause of death was septic shock due to a pseudomonas infection which first compromised the urinary tract and the lungs. The man was 183 cm high and weighted 104 kg with a BMI of 31.19 kg/m2. He was a heavy smoker and an occasional alcohol drinker. He had pre-existing cerebrovascular, cardiovascular, and respiratory chronic diseases. Over his life he underwent minor interventions (melanoma removal, cataract surgery). Additionally, he had an untreated umbilical hernia. The presence of comorbidities could limit the generalizability of the model. However, the reported medical history has a limited effect on vertebral tissues.

The fresh-frozen cadaver was prepared with the aim of advanced surgical education in surgery of the spine, using the best standards of practice to preserve normal spine anatomy. Imaged without equipment and/or clothing, at room temperature.

### Ethical compliance

The cadaver was obtained from MedCure Inc., guarantor of clinical data of cadaver donor. The donor application form was signed willingly to science before death by the donor itself. The document reported the possibility of deriving data and models for scientific research. Medcure was informed about this study and gave the consent for dataset publication. In accordance, the IRCCS Humanitas Research Hospital - Data Protection Office agreed in allowing the publication for human data. The IRCCS Humanitas Research Hospital - Ethics Committee agreed on the study and the publication of the data.

### Image acquisition protocol

Computed tomography (CT) acquisitions of the cadaveric trunk were performed on 3 different CT scanners: a. Revolution CT (GE HealthCare, 256 slices, defined as Scanner 1); b. Revolution EVO (GE HealthCare, 64 slices, defined as Scanner 2); c. Ingenuity CT (Philips Healthcare, 64 slices, defined as Scanner 3).

We performed a Test-Retest protocol on a single scanner (Scanner 1) to assess intrascanner repeatability of RFs. Retest acquisition was performed 1 h after Test acquisition, upon cadaver repositioning.

The complete acquisition protocol was structured with respect to 2 main sequences:

- KV variable: the acquisitions were performed at 300 mA, changing the kV parameter from 80 kV to 140 kV with 20 kV steps.
- mA variable: the acquisitions were performed at 120 KV, changing the mA parameter from 250 mA to 400 mA with 50 mA steps.

**Fig. 1:**
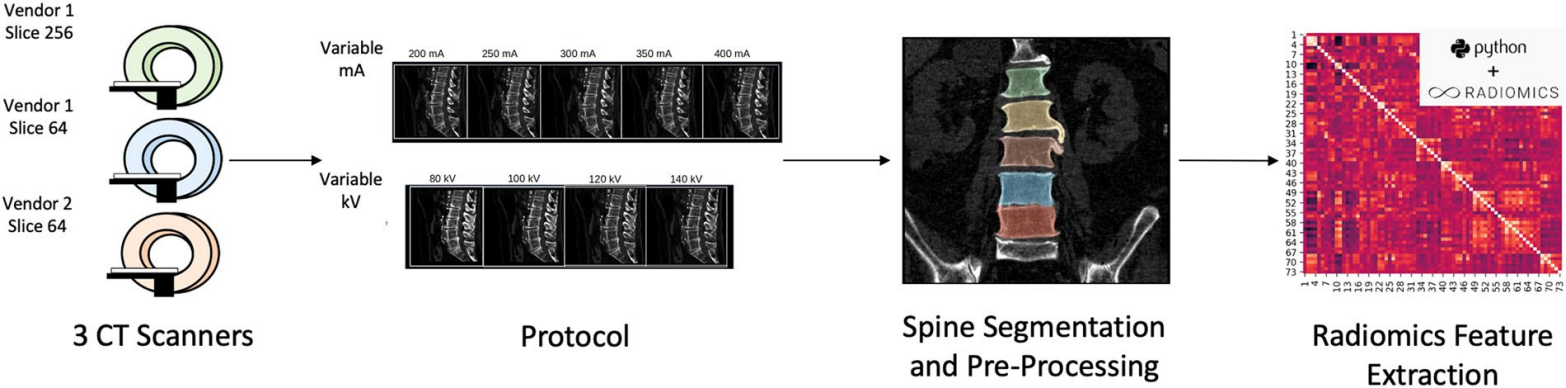
Study design. The images were acquired on 3 CT scanners defined as Scanner 1, Scanner 2 and Scanner 3. KV and mA variation protocols were acquired, and lumbar vertebrae were segmented through convolutional neural network. Following, radiomics features were extracted using pyradiomics library.

Each sequence was acquired using two fields of view (FOV): Abdomen (500 mm) and Spine (320 mm). Each volume was reconstructed with both the Standard Soft Tissue Kernel and the Bone Kernel. Thus, we obtained 36 unique CT datasets for each complete protocol. Specifically, 50 unique volumes (36 for protocol plus 14 for Retest dataset) were acquired on Scanner 1, 26 volumes (10 Abdomen FOV missing due to overheating of the x-ray tube) were obtained on Scanner, 36 CT volumes were acquired on Scanner 3, resulting in a total of 112 acquisitions. The complete protocol was acquired in one single session with a total acquisition time of 45 minutes, and is reported in Fig. 1.

### Spine segmentation

**Fig. 2:**
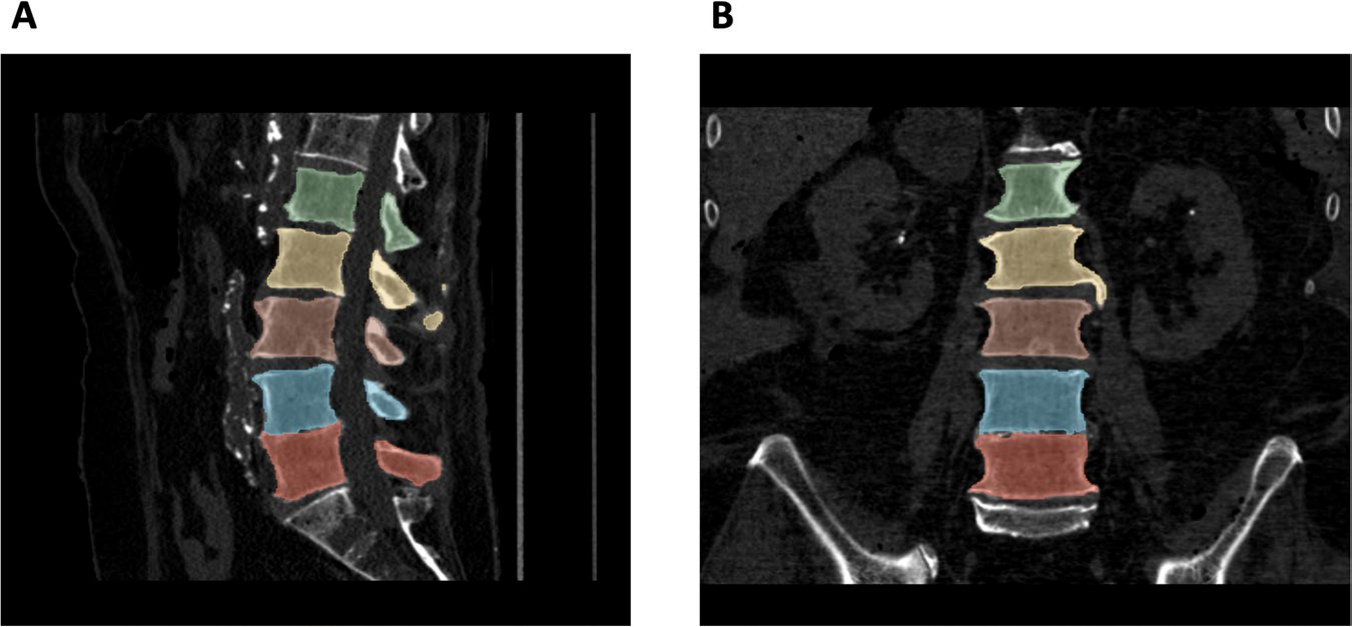
Deep Learning Segmentation of Lumbar Vertebrae. Volumetric segmentation of lumbar vertebrae shown in sagittal (**A**) and coronal (**B**) views. Each lumbar vertebra was assigned with a unique color (L1-green, L2-yellow, L3-brown, L4-blue, L5-red).

The lumbar vertebrae from each CT dataset were automatically segmented using a convolutional neural network (CNN) with nnU-Net structure^13^, which had been fine-tuned through a Transfer Learning procedure on an internal cohort of 180 lumbosacral CT images acquired in our Institution on different patients on 5 CT scanners (including the 3 scanners employed in this study)^14^. Each segmentation was manually checked and modified by two experienced neuroradiologists (in consensus). A representative image of spine segmentation is reported in Fig. 2.

### Radiomics feature extraction

RFs were extracted using the pyradiomics library (version 3.0.1), a software adhering to the Image Biomarker Standardization Initiative (IBSI) protocol^4^ and the steps were performed in suggestion to van Timmeren *et al*.^15^ recommendation. Image segmentation was performed using the above-mentioned deep-learning based U-Net and the features were extracted from a composite volume of interest (VOI) formed by the union of all the lumbar vertebrae VOIs. Image processing steps included only setting the bin width to 15 HU, to preserve the effects due to the single acquisition parameters. No filters were applied to the original images. We extracted a total of 107 RFs on each VOI, divided into 14 shape features, 18 first order features, 24 grey level co-occurrence matrix (GLCM) features, 16 grey level run length matrix (GLRLM) features, 16 grey level size zone matrix (GLSZM) features, 14 grey level dependence matrix (GLDM), and 5 neighboring gray-tone difference matrix (NGTDM).

## Data Records

The complete dataset of 112 CT images obtained with 3 different CT scanners, tube current and voltage, kernel reconstruction and field of view are available at the following Zenodo repository^16^.

The repository consists of a folder “Images” including all the CT images saved as NifTi files. All files are named in the following format: “CTscanner_TubeCurrent_TubeVoltage_KernelReconstruction_FieldOfView_Protocol.nii.gz”.

The folder “Segmentation” contains the complete set of lumbar vertebrae segmentations segmented by 3D CNN, which are named as the corresponding CT image.

## Technical Validation

All the 3 CT scanners are subject to regular maintenance and calibration, and CT acquisition protocols were set by dedicated radiographer with 25 years of experience.

Image segmentations were checked and modified in consensus by two experienced neuroradiologists.

**Fig. 3:**
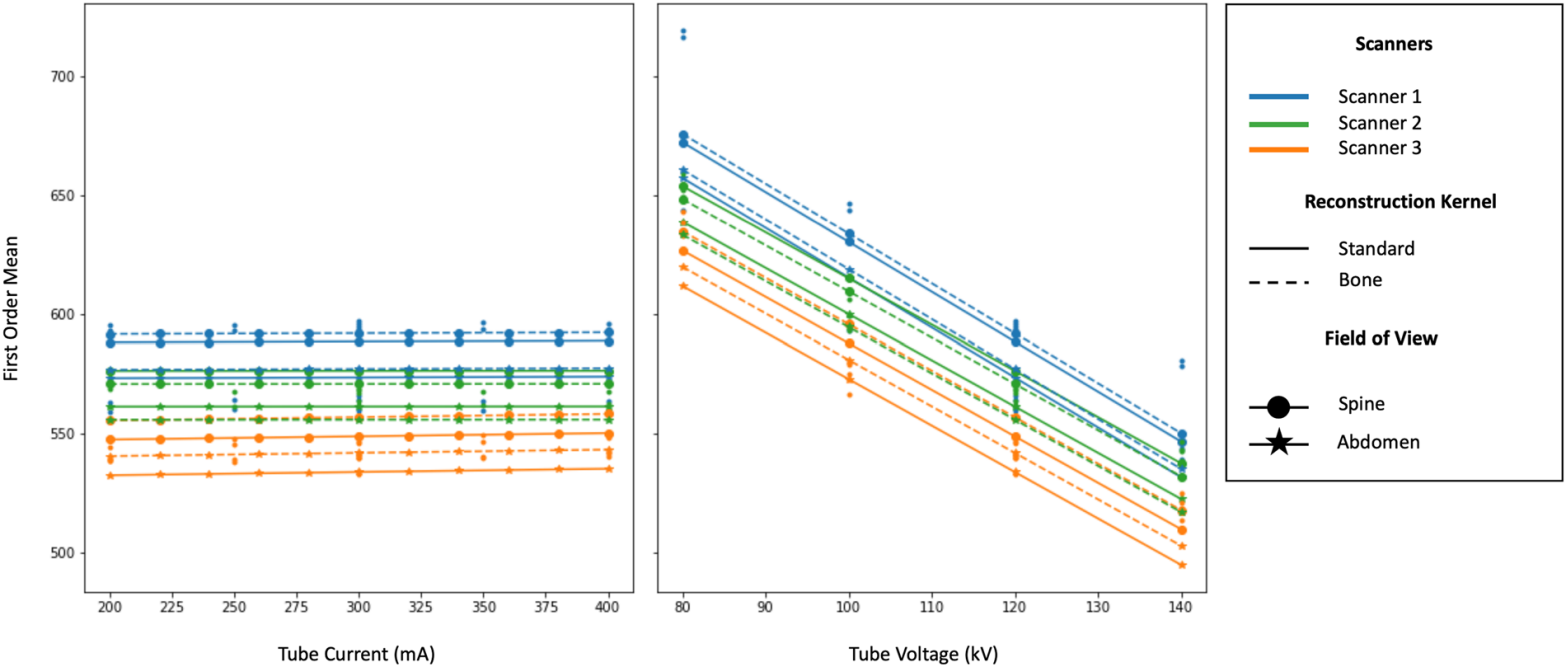
Generalized Linear Model representation of a Radiomics Feature for First Order family (Mean). Left panel represents the relation between Radiomics Feature and Tube Current in respect to Scanner, Reconstruction Kernel and Field of View. Right panel represents the relation between Radiomics Feature and Tube Voltage in respect to Scanner, Reconstruction Kernel and Field of View.

Further, we evaluated the effects of CT acquisition protocols in terms of mean Hounsfield Unit inside the vertebral bodies. The “First Order Mean” radiomics feature was employed for this purpose. A multivariate generalized linear model was computed to evaluate the effects of tube current and voltage, CT scanner, kernel reconstruction and field of view (see Fig. 3). The model was computed using a Gaussian link function as implemented by statsmodels library (v. 0.14.1).

Variations on tube current results in a constant relationship (p = 0.945), whereas tube voltage in an inverse linear relationship (p < 0.001). Scanner 2 was not statistically different in respect to Scanner 1 (p = 0.493), whereas Scanner 3 it is statistically different from Scanner 1 (p = 0.043). There is no difference between reconstruction kernel (p = 0.578) and field of view (p = 0.488).

## Usage Notes

The dataset will be provided inside the Zenodo repository (https://zenodo.org/records/10053317)^16^ by including a brief description of the data usage.

All CT images and relative segmentations are saved in NifTi format and could be visually inspected with several open-source viewers for medical images (including 3D Slicer, ITK-SNAP,…). Radiomics feature extraction should be performed with software that adhere with IBSI standards to assure reproducibility.

### Limitations

Some limitations are present in the dataset. Firstly, the dataset comprises the acquisition of a single subject, thus limiting possible inter-subject variability assessment. Secondly, soft tissue morphology could be altered due to the lack of blood flow. Thirdly, the underlying disease mentioned in “Cadaver donor” paragraph could have some marginal impact on vertebrae analysis, but higher impact on surrounding organs.

## Data Availability

Code for Radiomics feature extraction and GLM model is included in the Zenodo reposity of the dataset^16^.
